# ChREBP Rather Than SHP Regulates Hepatic VLDL Secretion

**DOI:** 10.3390/nu10030321

**Published:** 2018-03-07

**Authors:** Hiroyuki Niwa, Katsumi Iizuka, Takehiro Kato, Wudelehu Wu, Hiromi Tsuchida, Ken Takao, Yukio Horikawa, Jun Takeda

**Affiliations:** 1 Department of Diabetes and Endocrinology, Graduate School of Medicine, Gifu University, Gifu 501-1194, Japan; hiroyu760202@yahoo.co.jp (H.N.); bado_aberu@yahoo.co.jp (T.K.); wudelehu100@126.com (W.W.); Gif095@gifu-u.ac.jp (H.T.); lamgerrpard@yahoo.co.jp (K.T.); yhorikaw@gifu-u.ac.jp (Y.H.); jtakeda@gifu-u.ac.jp (J.T.); 2 Gifu University Hospital Center for Nutritional Support and Infection Control, Gifu 501-1194, Japan

**Keywords:** carbohydrate response element binding protein, small heterodimer partner, microsomal triglyceride transfer protein, very-low-density lipoprotein

## Abstract

The regulation of hepatic very-low-density lipoprotein (VLDL) secretion plays an important role in the pathogenesis of dyslipidemia and fatty liver diseases. VLDL is controlled by hepatic microsomal triglyceride transfer protein (MTTP). *Mttp* is regulated by carbohydrate response element binding protein (ChREBP) and small heterodimer partner (SHP). However, it is unclear whether both coordinately regulate *Mttp* expression and VLDL secretion. Here, adenoviral overexpression of ChREBP and SHP in rat primary hepatocytes induced and suppressed *Mttp* mRNA, respectively. However, *Mttp* induction by ChREBP was much more potent than suppression by SHP. Promoter assays of *Mttp* and the liver type pyruvate kinase gene revealed that SHP and ChREBP did not affect the transcriptional activity of each other. *Mttp* mRNA and protein levels of Shp^−/−^ mice were similar to those of wild-types; however, those of Chrebp^−/−^Shp^−/−^ and Chrebp^−/−^ mice were significantly much lower. Consistent with this, the VLDL particle number and VLDL secretion rates in Shp^−/−^ mice were similar to wild-types but were much lower in Chrebp^−/−^ and Chrebp^−/−^Shp^−/−^ mice. These findings suggest that ChREBP, rather than SHP, regulates VLDL secretion under normal conditions and that ChREBP and SHP do not affect the transcriptional activities of each other.

## 1. Introduction

Nonalcoholic fatty liver disease (NAFLD) is the most common chronic liver disease in the world [1,2]. High fat diets and high carbohydrate diets are responsible in the development of NAFLD [3,4,5]. High carbohydrate diets cause fatty liver by increasing hepatic de novo lipogenesis [3]. High fat diets increase hepatic fat content [4]. Therefore, lifestyle modifications, including hypocaloric diets, are critical for the prevention and treatment of NAFLD. Non-alcoholic fatty liver disease (NAFLD) is associated with hepatic insulin resistance and hepatic fibrosis and can lead to the development of diabetes mellitus, dyslipidemia, non-alcoholic steatohepatitis, and hepatocellular carcinoma [1,2]. Hepatic lipid accumulation is regulated by the free fatty acid (FFA) supply from adipose tissue, and de novo lipogenesis from the secretion of glucose, acyl CoA oxidation, and very-low-density lipoprotein (VLDL) in the liver [6]. Visceral adipose tissue is the primary source of hepatic fat in adults, contributing 59% of the triglycerides found in the liver [6]. Glucose and insulin signals regulate hepatic de novo lipogenesis through the carbohydrate response element binding protein (ChREBP) and sterol response element binding protein 1c (SREBP1c), respectively [7]. Moreover, altered VLDL secretion also contributes to the pathogenesis of NAFLD [8,9]. VLDL secretion is controlled by microsomal triglyceride transfer protein (MTTP) [8,9]. MTTP is essential for the assembly and secretion of apolipoprotein B-containing lipoproteins. In humans and mice, *Mttp* deficiency causes hypolipidemia and fatty liver [8,9]. Thus, *Mttp* is involved in the pathogenesis of NAFLD.

*Mttp* regulation depends on a few highly-conserved *cis*-elements in its promoter. The *Mttp* promoter sequence contains critical positive (hepatic nuclear factor [HNF]-1, HNF-4, direct repeat 1, and FOX) and negative regulatory sterol and insulin response elements [8,9]. Small heterodimer partner (SHP, also known as NR0B2) is a unique nuclear receptor (NR) that contains the dimerization and ligand-binding domain found in other family members but lacks the conserved DNA-binding domain [10]. We previously reported that human *SHP* genetic variations appear to cause mild obesity and type 2 diabetes mellitus [11,12]. As a co-repressor, SHP represses the activities of HNF-4α and the retinoid X receptor liver receptor homolog-1 (LRH-1) by interacting with these factors [13,14]. However, it is not certain whether SHP affects the VLDL secretion rate [15,16]. 

As with the effects of nutritional conditions on Mttp expression, high fat diets and high carbohydrate diets induce Mttp expression, resulting in hyperlipidemia [17,18]. High fat diets increase hepatic *Mttp* expression through decreased binding of sterol regulatory element-binding proteins to the *Mttp* promoter [17]. High sucrose and high fructose consumption also increase Mttp expression [18]; however, the mechanisms of this are unclear. Recently, we reported that *Mttp* mRNA levels and VLDL secretion rates were lower in the livers of Carbohydrate Response Element Binding Protein [ChREBP] knockout (Chrebp^−/−^) mice [19]. ChREBP is a glucose-activated transcription factor that regulates hepatic de novo lipogenesis in the liver [20,21,22,23]. ChREBP transcriptional activities are regulated by phosphorylation/dephosphorylation, nuclear translocation, and conformational changes [20,21,22,23]. It was recently proposed that ChREBP transcriptional activity is regulated through interactions with nuclear factors, such as farnesoid X receptor (FXR) and HNF4a [24,25,26,27,28]. Moreover, computer analysis revealed that ChREBP contains a nuclear receptor binding motif [29]. Considering that SHP represses the activities of many transcription factors by interacting with these factors [10], we speculated that SHP might also affect *Mttp* transcription in cooperation with ChREBP.

ChREBP and SHP control the regulation of *Mttp* expression. Using rat hepatocytes and knockout mice, we therefore evaluated the following: (1) whether ChREBP and SHP affect the transcriptional activities of each other; (2) whether ChREBP and SHP coordinately affect *Mttp* expression and thereby VLDL secretion; and (3) if ChREBP or SHP regulate VLDL secretion more potently. An appreciation of the roles of ChREBP and SHP in regulating *Mttp* and VLDL secretion will be beneficial for understanding the role of nutritional signals in the development of NAFLD and dyslipidemia. 

## 2. Materials and Methods 

### 2.1. Establishment of Chrebp^−/−^ Shp^−/−^ Double Knockout (DKO) Mice

Animal experiments were carried out in accordance with the National Institute of Health Guide for the Care and Use of Laboratory Animals (NIH Publications No. 8023, revised 1978). All animal care was approved by the animal care committee of the University of Gifu (No. 27-30, approval date: 4 June 2015). Mice were housed at 23 °C on a 12-h light/dark cycle. Chrebp^−/−^ mice were backcrossed for at least 10 generations onto the C57BL/6J background [19,30]. Shp^−/−^ mice were purchased from Lexicon Genetics Inc. (The Woodlands, TX, USA). Shp^+/−^ mice were backcrossed for at least 12 generations onto the C57BL/6J background. Male mice were used for all studies. Chrebp^−/−^Shp^−/−^ (DKO) mice were intercrossed with Chrebp^−/−^ and Shp^−/−^ mice. 

Mice had free access to water and were fed an autoclaved CE-2 diet (CLEA Japan, Tokyo, Japan). Wild-type (WT), Chrebp^−/−^, Shp^−/−^, and DKO mice were housed separately with a total of three mice per cage. Body weight was measured weekly between 7 and 21 weeks of age. Mice were sacrificed at 21 weeks of age by cervical dislocation. All tissue samples were immediately placed into liquid nitrogen and stored at −80 °C until further analysis for hepatic triacylglycerol and cholesterol contents and for quantitative polymerase chain reaction (PCR).

### 2.2. Liver Triglyceride and Cholesterol Content and Plasma Profile Measurements

Liver lipids were extracted using the Bligh and Dyer method [31]; they were measured using triglyceride (Wako Pure Chemicals, Osaka, Japan) and cholesterol E-tests (Wako). Blood plasma was collected from the retro–orbital venous plexus, following ad libitum feeding or after a 6-h fast. Blood glucose and beta-hydroxybutyrate (β-OHB) levels were measured using a FreeStyle Freedom monitoring system (Nipro, Osaka, Japan). Plasma insulin, FFA, fibroblast growth factor 21 (FGF21), triglyceride, and total cholesterol levels were determined using commercial assay kits, as follows: mouse insulin enzyme-linked immunosorbent assay (ELISA) (H type) (Shibayagi, Gunma, Japan), NEFA C-test (Wako Pure Chemicals, Tokyo, Japan), mouse/rat Fgf21 ELISA (R&D Systems, Minneapolis, MN, USA), triglyceride E-test (Wako), and the cholesterol E-test (Wako), respectively.

### 2.3. RNA Isolation and Quantitative Real-Time PCR

Total RNA isolation, cDNA synthesis, and real-time PCR analysis were performed as previously described [13]. Real-time PCR primers for mouse/rat Chrebp, liver type pyruvate kinase (Pklr), Fgf21, Mttp, and RNA polymerase II (Pol2) have been previously reported [31,32,33]. All amplifications were performed in triplicate. The relative amounts of mRNA were calculated using the comparative CT method. Pol2 expression was used as an internal control.

### 2.4. VLDL Secretion Test and MTTP Protein Contents

VLDL secretion tests were performed as previously reported [19]. Briefly, 500 mg/kg body weight tyloxapol was administered intraperitoneally to 5 h fasted mice. Blood sampling was performed at the indicated times. The triglyceride (TG) content in lipoprotein fractions and the VLDL particle numbers were analyzed using gel-permeation high-performance liquid chromatography (LipoSEARCH^®^) at Skylight Biotech Inc. (Akita, Japan) [34]. Serum samples obtained from six mice after 6 h fasting were pooled and measured. Liver MTTP protein contents were measured by an MTTP ELISA kit (Cloud-Clone Corp Inc., Houston, TX, USA). 

### 2.5. Adenoviral Delivery into Rat Primary Hepatocytes

Rat primary hepatocytes were isolated as previously described [32,33]. Isolated hepatocytes were suspended in DMEM, supplemented with 10% fetal calf serum, 100 nM insulin, 100 nM dexamethasone, 10 nM triiodothronine (T3), and 100 μg/mL penicillin/streptomycin. Cells were seeded in 6-well plates or 10-cm dishes and grown in a humidified atmosphere of 5% CO_2_ and 95% air at 37 °C. After the cells had been incubated for 4 h, the medium was replaced with DMEM containing 10 nM T3. Adenoviruses harboring dominant active rat Chrebp lacking 1–196 a.a. (Ad-daChREBP) and mouse SHP full length (Ad-SHP) were constructed according to the manufacturer’s protocol (Invitrogen, Carlsbad, CA, USA). After 4 h infection with Ad-daChREBP and/or Ad-SHP, rat hepatocytes were incubated for 20 h. *Mttp* mRNA levels were detected by real-time PCR. 

### 2.6. Transfections and Luciferase Reporter Assay

Reporter plasmids pGL3 3×LPK ChoRE, pcDNA-daChrebp, and pcDNA-empty have been previously reported [32,33]. pcDNA-SHP and pcDNA-FXR were constructed using the pcDNA™3.2-DEST Mammalian Expression Vector (Invitrogen) according to the manufacturer’s protocol (Invitrogen). pGL3-Mttp (−211 bp) was constructed as follows: mouse Mttp promoter regions (−211 bp to +77 bp) amplified by PrimSTAR MAX DNA Polymerase (Takara Bio, Kusatsu, Japan) were inserted into the pGL3 basic vector (Promega, Madison, WI, USA). Then, 1.0 μg of pGL3 MTTP (−211 bp), 0.1 μg pRL-TK vector, pcDNA-empty + pcDNA-daChREBP + pcDNA-SHP (total 1.0 μg), and 3 μL of Lipofectamine 2000 (Invitrogen) were transfected into primary rat hepatocytes. After incubation for 24 h, cells were collected and used in luciferase assays. 

### 2.7. Statistical Analysis

All values are presented as means ± standard deviations. Data were analyzed using Tukey’s test. A *p*-value < 0.05 was considered to be statistically significant.

## 3. Results

### 3.1. Adenoviral Overexpression of ChREBP and SHP Respectively Increased and Decreased Mttp Expression

**Figure 1:**
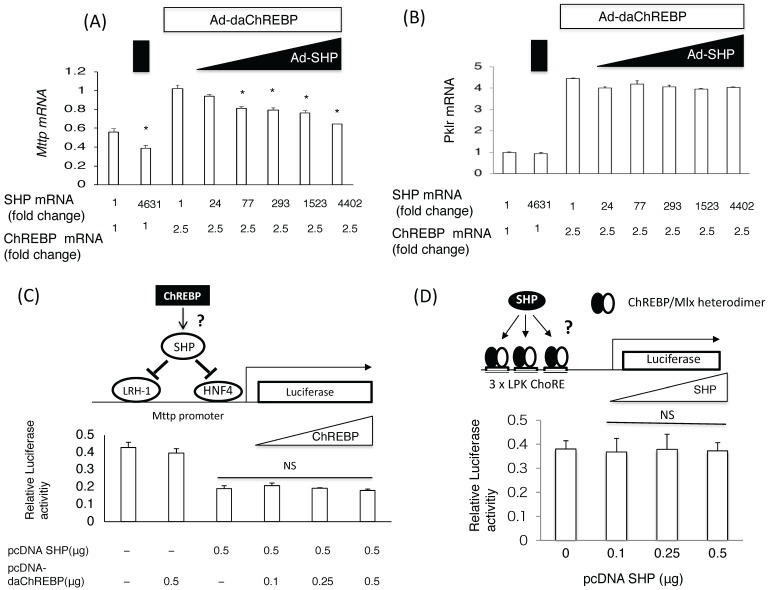
The coordinated effects of carbohydrate response element binding protein (ChREBP) and small heterodimer partner (SHP) on *Mttp* expression. (**A**) and (**B**) The effects of SHP on ChREBP-mediated *Mttp* (**A**) and *Pklr* (**B**) mRNA induction. After 4 h infection with Ad-daChREBP and/or Ad-SHP in hepatocytes, cells were incubated for 20 h. *Mttp* mRNA levels were detected by real-time Polymerase chain reaction method. Open symbols and closed symbols indicated adenoviral overexpression of daChREBP and SHP, respectively. The *x*-axis indicates *Shp and Chrebp* mRNA levels (fold change). Adenoviral overexpression of ChREBP caused a 2.5-fold increase in Chrebp mRNA levels. Adenoviral SHP expression caused an indicated fold increase in Shp expresssion. Pol2 expression was used as an internal control. *n* = 4 per group. * *p* < 0.05; (**C**) Reporter assay using pGL3-Mttp (−211 bp). The indicated amounts of pcDNA-daChREBP and pcDNA-SHP were transfected with pGL3-Mttp (−211 bp) and pRL-TK vectors and Lipofectamine 2000 reagent into primary rat hepatocytes. After 24 h incubation, cells were collected for the dual luciferase assay. Luciferase activity was normalized to Renilla luciferase activity. N.S., not significant. *n* = 6 per group; (**D**) Reporter assay using pGL3 3×LPK ChoRE. The indicated amount of pcDNA-SHP was cotransfected with pGL3-3×LPK ChoRE, PRL-TK, pcDNA daChREBP, and Lipofectamine 2000 into primary rat hepatocytes. After 24 h incubation, cells were collected for the dual luciferase assay. Luciferase activity was normalized to Renilla luciferase activity. NS, not significant vs control. *n* = 6 per group.

Adenoviral ChREBP caused an increase in *Mttp* and *Pklr* mRNA expression, while SHP suppressed only *Mttp* expression in primary rat hepatocytes (Figure 1A,B). Moreover, overexpressing SHP by more than 70-fold (above physiological levels) was found to suppress ChREBP-mediated *Mttp* induction (Figure 1A). In contrast, SHP overexpression failed to suppress ChREBP-mediated *Pklr* induction (Figure 1B). This suggested that ChREBP induced *Mttp* expression more potently than SHP suppressed it.

SHP is known to suppress HNF4/HNF1/LRH-1-mediated *Mttp* expression [13,35]. Accordingly, transfection of the PGL3 basic vector containing 211 bp of the *Mttp* promoter region (pcDNA-SHP), including the HNF4/HNF1/LRH-1 binding site, successfully suppressed PGL3-*Mttp* (−211 bp) luciferase activity. However, co-transfection of pcDNA-daChREBP did not reverse this effect (Figure 1C). In contrast, the transfection of the pcDNA SHP vector could not suppress pGL3 *Pklr* luciferase activities induced by pcDNA-daChREBP (Figure 1D). This suggested that SHP and ChREBP did not affect the transcriptional activities of each other

### 3.2. Chrebp Shp DKO Mice Resembled Chrebp^−/−^ Mice

We next assessed the effects of ChREBP and SHP on the mouse metabolic phenotype in vivo. *Chrebp^−/−^* mice displayed characteristically higher liver weights, lower white adipose tissue weights, higher plasma FFA, FGF-21, and β-OHB levels, and elevated liver glycogen contents compared with those of WT mice. Chrebp^−/−^Shp^−/−^ double knockout (DKO) mice also displayed similar characteristics (Table 1).

**Figure 2:**
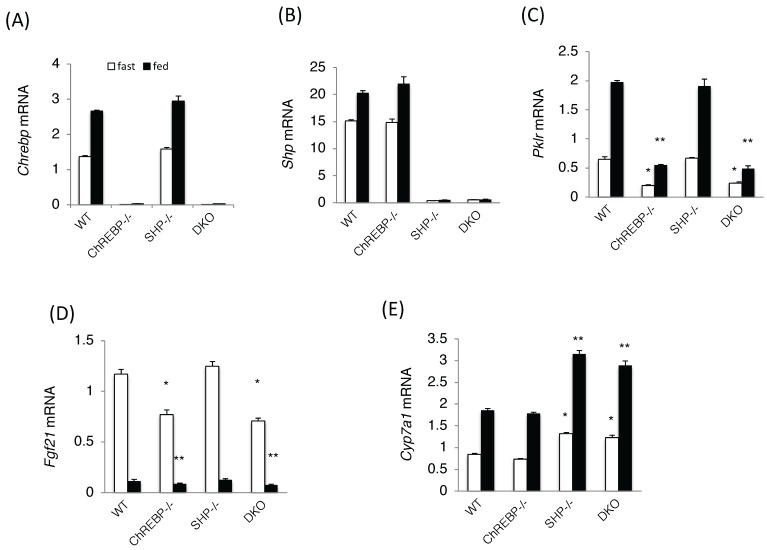
ChREBP and SHP target mRNA levels in wild-type, Chrebp^−/−^, Shp^−/−^, and Chrebp^−/−^ Shp^−/−^ mice. Carbohydrate response element binding protein (Chrebp) (**A**); small heterodimer partner (Shp) (**B**); liver type pyruvate kinase (Pklr) (**C**); fibroblast growth factor 21 (Fgf21) (**D**); and Cholesterol 7 alpha-hydroxylase (Cyp7a1); (**E**) mRNA expression analysis in the livers of wild-type, Chrebp^−/−^, Shp^−/−^, and Chrebp^−/−^ Shp^−/−^ (DKO) mice. Open squares indicate fasted conditions; closed squares indicate fed conditions. Pol2 expression was used as an internal control. Each mRNA levels were corrected with mouse RNA polymerase 2 mRNA. * *p* < 0.05 vs. WT (fast), *n* = 4. ** *p* < 0.05 vs. WT (fed), *n* = 4.

*Chrebp* and *Shp* mRNA levels were unaffected by *Shp* and *Chrebp* deletions, respectively (Figure 2A,B). Consistent with these phenotypes, the expression levels of ChREBP target genes, such as *Pklr* and *Fgf21*, were significantly lower in Chrebp*^−/−^*and DKO mice (Figure 2C,D). In contrast, the expression of *Cyp7a1*, a SHP target gene, was significantly higher in Shp^−/−^ and DKO mice but was unchanged in Chrebp*^−/−^* mice (Figure 2E). Many phenotypes seen in DKO mice were similar to those of Chrebp^−/−^ mice, suggesting that SHP had little effect on ChREBP target gene expression. 

### 3.3. ChREBP and SHP Respectively Positively and Negatively Controlled VLDL Secretion through Mttp Regulation

**Figure 3:**
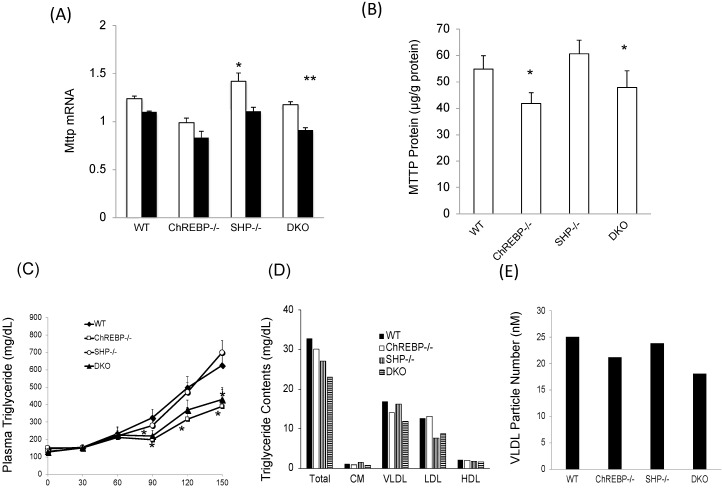
Shp deletion failed to recover decreased Mttp expression, and thereby, VLDL secretion rates mediated by Chrebp deletion. (**A**) Hepatic *Mttp* mRNA levels in the livers of wild-type (WT), Chrebp^−/−^, Shp^−/−^, and DKO mice. Mttp mRNA levels were detected by real-time PCR. Pol2 expression was used as an internal control. * *p* < 0.05 vs. WT (fast), *n* = 4. ** *p* < 0.05 vs. WT (fed), *n* = 4. Data are represented as means ± SD; (**B**) Hepatic MTTP protein expression in the livers of WT, Chrebp^−/−^, Shp^−/−^, and DKO mice. MTTP protein levels were measured by ELISA and corrected for total protein levels. * *p* < 0.05 vs. WT, *n* = 5–7. Data are represented as means ± SD; (**C**) VLDL secretion rates were measured as previously reported [17]. * *p* < 0.05 vs. WT, *n* = 5–7. Data are represented as means ± SD; TG contents in lipoprotein fraction (**D**) and VLDL particle numbers (**E**) were analyzed using gel-permeation high-performance liquid chromatography. Data are representative of six samples pooled.

We evaluated the effects of *Chrebp* and *Shp* deletion on *Mttp* expression. Hepatic *Mttp* mRNA levels were much lower in Chrebp^−/−^ and DKO mice than in WT mice; however, those in Shp^−/−^ mice were only slightly (only 1.15 times) higher than in WT mice (Figure 3A). Consistent with mRNA levels, MTTP protein levels were significantly much lower in Chrebp^−/−^ and DKO mice than in WT mice (Figure 3B). Moreover, VLDL TG contents of Chrebp*^−/−^* and DKO mice were lower than those in WT and Shp^−/−^ mice, while VLDL secretion rates in Chrebp^−/−^ and DKO mice were approximately 0.6 times lower than in WT mice (Figure 3C). In contrast, liver VLDL secretion rates in Shp*^−/−^* mice were similar to those in WT mice (Figure 3C). In support of this, the VLDL TG contents and VLDL particle numbers in Chrebp*^−/−^* mice and DKO mice were much lower those in WT mice, while those in Shp*^−/−^* mice were similar to those in WT mice (Figure 3D,E). Therefore, under normal conditions, the effect of ChREBP on *Mttp* expression was potent but the effect of SHP was physiologically much weaker or lacking.

## 4. Discussion

In this study, we evaluated whether ChREBP and SHP could coordinately affect VLDL secretion via *Mttp* expression. ChREBP and SHP reciprocally affected *Mttp* mRNA levels; however, the potency of *Mttp* suppression by SHP was much lower than that of *Mttp* induction by ChREBP. Therefore, ChREBP and SHP did not affect the transcriptional activity of each other. Mttp mRNA and protein levels in Chrebp^−/−^ and DKO mice were much lower than those in WT mice; however, those in Shp^−/−^ mice were similar to WT. In agreement with this, VLDL secretion rates of DKO and Chrebp^−/−^ mice were much lower than those of Shp^−/−^ mice, which were the same as those of WT mice. Together, these findings suggest that ChREBP, rather than SHP, regulates *Mttp* expression under normal conditions.

SHP predominantly functions as a transcriptional repressor of gene expression that binds directly to nuclear receptors, such as LRH-1, HNF4α, estrogen receptors, estrogen receptor-related receptors, liver X receptors, peroxisome proliferator-activated receptors, glucocorticoid receptor, thyroid hormone receptor β, retinoic acid receptor α, FXR, pregnane X receptor, constitutive androstane receptor, androgen receptor, nerve growth factor IB, and common heterodimerization partner retinoid X receptors [10]. SHP was previously shown to suppress MTTP expression by binding to HNF4α/LRH-1 sites in the *Mttp* promoter [10,13,35]. 

Recent reports have proposed a new ChREBP regulatory mechanism (ChREBP-nuclear receptor interaction), in which nuclear factors interact with ChREBP to modify ChREBP transcriptional activity [25,26,27,28]. Computer analysis previously revealed that ChREBP contains an LxQLLT sequence that matches the NR binding motif [29]. The rat LxQLLT sequence is localized to a proline-rich region (540–545 a.a.) of ChREBP [29]. SHP binds and represses the transcriptional activities of target genes by utilizing two functional LXXLL-related motifs located in the ligand-binding domain [10]. Some studies have also suggested that the interactions between ChREBP and nuclear receptors, such as FXR and HNF4, play physiological roles in regulating *Pklr* expression [25,26,27,28]. HNF4 and FXR positively and negatively regulate ChREBP transcriptional activity, respectively. However, our data revealed that SHP overexpression did not affect *Pklr* mRNA or luciferase activity of the PGL3 3xPKLR ChoRE vector. Moreover, *SHP* deletion did not affect the mRNA levels of ChREBP target genes (*Pklr* and *Fgf21*) in mice. Consistent with this, we found that ChREBP interacted with SHP in the mammalian two-hybrid system, and interactions with SHP did not interfere with the binding between FXR and ChREBP in the mammalian two-hybrid system (data not shown). This indicated that SHP might not affect the interplay between ChREBP and nuclear factors such as FXR and HNF4, and therefore, that it does not modulate ChREBP transcriptional activity.

Reporter assays for the *Mttp* promoter (−211/+81 bp) in the present study revealed that SHP suppressed hepatic *Mttp* expression, but that ChREBP overexpression did not affect luciferase activity controlled by the *Mttp* promoter (−211/+81 bp). This supports the notion that ChREBP does not affect the role of SHP as a corepressor. Taken together with the fact that a *Chrebp* deletion did not affect SHP target gene (*Cyp7a1*) mRNA levels, this suggested that ChREBP does not modulate SHP transcriptional activity. Thus, ChREBP and SHP do not affect the transcriptional activities of each other.

To evaluate ChREBP binding to *Mttp* promoter regions, we searched for putative ChoRE motifs [36,37,38] in the promoter, exons, and introns. However, we did not detect ChoREs in *Mttp*. Considering that ChREBP regulates the expression of many genes [21,30,32,33,36,37,38,39], we propose that it might indirectly induce *Mttp* expression through the activation of other transcription factors. However, further investigation is needed to identify the mechanism underlying the ChREBP induction of *Mttp* expression.

The VLDL secretory pathway is regulated by MTTP, a rate-limiting enzyme. We previously reported that ChREBP positively regulates VLDL secretion [19]. Although SHP controls *Mttp* expression by modulating HNF4 and LRH-1 transcriptional activity, its effect on VLDL secretion was controversial [15,16]. Some studies reported that VLDL secretion in Shp^−/−^ mice on ob/ob or C57BL/6 backgrounds increased relative to WT mice [16], while others showed that VLDL secretion in Shp^−/−^ mice fed a Western diet was similar to that in Shp^−/−^ mice fed a normal diet [15]. Our data were consistent with the latter. They also confirmed that ChREBP regulates VLDL secretion, but that *SHP* deletion did not affect VLDL secretion in either WT or Chrebp^−/−^ mice. Our analyses of MTTP protein levels and VLDL particle numbers also supported these data. Taken together with the fact that SHP overexpression by more than 70 times was needed to suppress ChREBP-mediated *Mttp* induction, these findings suggest that ChREBP plays important roles in VLDL secretion, while those of SHP are much smaller or negligible.

**Figure 4:**
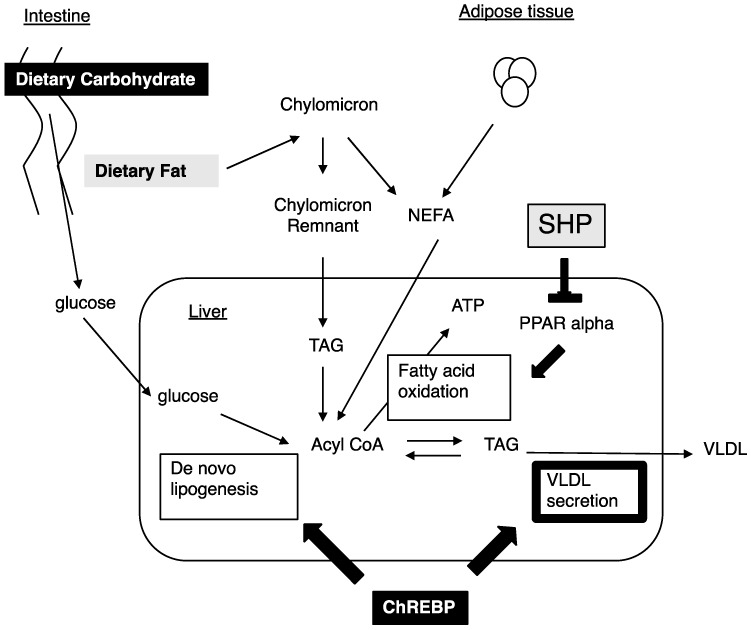
ChREBP and SHP independently regulate hepatic lipid metabolism. High carbohydrate diets increase de novo lipogenesis and VLDL secretion through ChREBP activation. Therefore, Chrebp deletion prevents high carbohydrate diet induced fatty liver [30,40,42,43]. In contrast, SHP suppresses PPAR alpha and an Shp deletion prevents high fat diet induced fatty liver [15,45]. In this study, we clarified that ChREBP, rather than SHP, regulates VLDL secretion through *Mttp* expression (bold line). Thus, ChREBP and SHP independently regulate hepatic lipid metabolism.

Nutritional conditions affect the development of NAFLD and dyslipidemia. High carbohydrate diets and high fat diets increase Mttp expression [9]. High carbohydrate diets increase de novo lipogenesis and *Mttp* expression [9,18]. As we previously reported, Chrebp deletions prevent hepatic lipid accumulation following consumption of a high carbohydrate diet [30,40]. The prevention from high carbohydrate diet induced fatty liver was due to a decrease in de novo lipogenesis, decreased free fatty acid supply from adipose tissue, and appetite loss from the high sucrose and high fructose diet [30,40,41,42,43]. Decreased *Mttp* expression might deteriorate NAFLD in ChREBP^−/−^ mice. As ChREBP^−/−^ mice could not be fed a high carbohydrate diet such as the high sucrose diet [30,41,42], we could not perform an experiment using the high carbohydrate diet. In contrast, a high fat diet increased the FFA supply from dietary fat and induced Mttp expression [17]. The High fat diet suppressed ChREBP transcriptional activity [44]. Moreover, Chrebp deletion did not prevent from high fat diet induced fatty liver owing to decreased fatty acid oxidation [19,43]. As Shp suppress PPAR alpha [45], Shp deletion prevented hepatic lipid accumulation through increased fatty acid oxidation [15]. Consistent with our data, *Mttp* expression was not affected by Shp deletion. Therefore, we analyzed the effects of ChREBP and SHP deletion on *Mttp* expression and VLDL secretion under a normal diet condition. In this study, ChREBP and SHP independently regulated glucose and lipid metabolism. The difference in target genes between ChREBP and SHP might contribute to the different effects of ChREBP and SHP on preventing the development of fatty liver induced by high fat diets in Chrebp^−/−^ and Shp^−/−^ mice (Figure 4). 

## 5. Conclusions

ChREBP and SHP were shown to modulate hepatic *Mttp* expression, but the capacity of *Mttp* suppression by SHP was much lower than that of *Mttp* induction by ChREBP. Moreover, ChREBP and SHP did not affect the transcriptional activities of each other. Unlike many transcription factors, ChREBP was not regulated by SHP. Finally, ChREBP, rather than SHP, was shown to regulate hepatic VLDL secretion. 

## Figures and Tables

**Table 1:** Phenotypic comparison between WT, ChREBP^−/−^, SHP^−/−^, ChREBP^−/−^SHP^−/−^ (DKO) mice.

	WT	ChREBP^−/−^	SHP^−/−^	DKO
BW (g)	29.50 ± 0.71	30.40 ± 1.92	28.57 ± 2.98	30.2 ± 2.51
Liver (%BW)	5.02 ± 0.08	5.37 ± 0.27 *	5.01 ± 0.14	5.52 ± 0.24 *
Epidydimal fat (%BW)	2.41 ± 0.30	1.68 ± 0.62 *	2.07 ± 0.33	1.41 ± 0.24 *
Mesenteric fat (%BW)	1.16 ± 0.13	0.77 ± 0.16 *	1.02±0.28	0.55 ± 0.11 *
Brown adipose tissue (%BW)	0.30 ± 0.07	0.27 ± 0.10	0.36 ± 0.12	0.49 ± 0.15
Plasma glucose (mg/dL)	132.3 ± 17.3	128.4 ± 13.2	118.7 ± 21.5	117.2 ± 11.5
Plasma insulin (ng/dL)	0.86 ± 0.26	1.01 ± 0.33	0.91 ± 0.41	0.65 ± 0.17
HOMA-R	0.28 ± 0.08	0.32 ± 0.12	0.27 ± 0.15	0.19 ± 0.03
Plasma triglyceride (mg/dL)	66.1 ± 10.1	56.2 ± 11.5	65.2 ± 12.6	67.3 ± 19.8
Plasma FGF21 (pg/mL)	802.1 ± 365.8	150.2 ± 51.1 *	584.1 ± 268.6	124.0 ± 61.8 *
Plasma β-hydroxybutyrate (mM)	2.05 ± 0.24	1.32 ± 0.11	2.37 ± 0.40	1.49 ± 0.22
Liver glycogen content (mg/g liver)	40.5 ± 12.4	80.8 ± 20.2 *	33.4 ± 10.5	78.0 ± 22.7 *
Liver cholesterol content (mg/g liver)	8.82 ± 1.63	9.54 ± 1.02	8.98 ± 1.53	7.17 ± 1.66
Liver triglyceride content (mg/g liver)	17.3 ± 4.2	16.0 ± 3.77	17.2 ± 5.14	15.7 ± 3.17

BW, body weight; EP, epidydimal fat weight; VS, visceral fat weight; BAT, brown adipose tissue weights; FGF21, fibroblast growth factor 21. * *p* < 0.05 vs. WT.
